# Collateral Projections Innervate the Mammillary Bodies and Retrosplenial Cortex: A New Category of Hippocampal Cells

**DOI:** 10.1523/ENEURO.0383-17.2018

**Published:** 2018-03-08

**Authors:** Lisa Kinnavane, Seralynne D. Vann, Andrew J. D. Nelson, Shane M. O’Mara, John P. Aggleton

**Affiliations:** 1 School of Psychology, Cardiff University, Cardiff, CF10 3AT, United Kingdom; 2 Trinity College Institute of Neuroscience, Trinity College, Dublin, D2, Ireland

**Keywords:** anterior thalamic nuclei, cingulate cortex, episodic memory, fornix, subiculum

## Abstract

To understand the hippocampus, it is necessary to understand the subiculum. Unlike other hippocampal subfields, the subiculum projects to almost all distal hippocampal targets, highlighting its critical importance for external networks. The present studies, in male rats and mice, reveal a new category of dorsal subiculum neurons that innervate both the mammillary bodies (MBs) and the retrosplenial cortex (RSP). These bifurcating neurons comprise almost half of the hippocampal cells that project to RSP. The termination of these numerous collateral projections was visualized within the medial mammillary nucleus and the granular RSP (area 29). These collateral projections included subiculum efferents that cross to the contralateral MBs. Within the granular RSP, the collateral projections form a particularly dense plexus in deep Layer II and Layer III. This retrosplenial termination site colocalized with markers for VGluT2 and neurotensin. While efferents from the hippocampal CA fields standardly collateralize, subiculum projections often have only one target site. Consequently, the many collateral projections involving the RSP and the MBs present a relatively unusual pattern for the subiculum, which presumably relates to how both targets have complementary roles in spatial processing. Furthermore, along with the anterior thalamic nuclei, the MBs and RSP are key members of a memory circuit, which is usually described as both starting and finishing in the hippocampus. The present findings reveal how the hippocampus simultaneously engages different parts of this circuit, so forcing an important revision of this network.

## Visual Abstract

**Figure:**
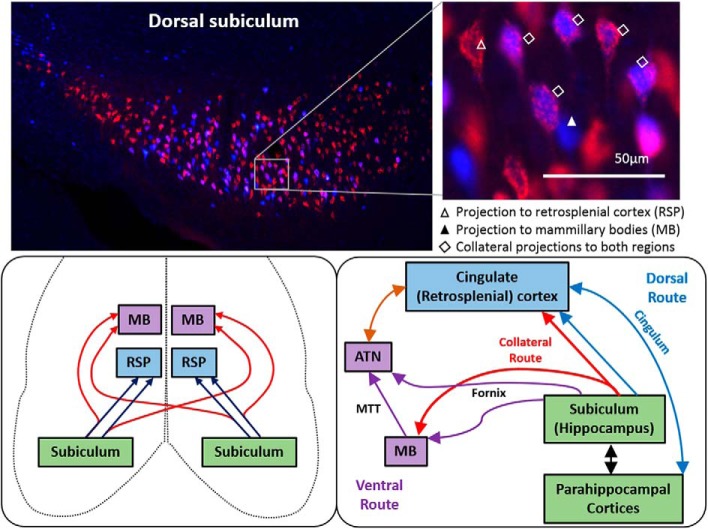

## Significance Statement

The hippocampus has both cortical and subcortical connections that are critical for spatial learning in rodents and episodic memory in humans. Chief among these connections are the dense hippocampal inputs to the retrosplenial cortex (RSP) and mammillary bodies (MBs), both of which originate in the subiculum. The present experiments reveal that in rodents approximately half of these retrosplenial projections have collaterals that also innervate the MBs. Consequently, these two areas share common hippocampal information, despite playing different roles in cognition. These same collateral projections contradict longstanding ideas about extended, serial hippocampal networks for memory. As these networks are affected from the earliest stages of Alzheimer’s disease, when memory disorders first appear, there is added significance in understanding their precise connectivity.

## Introduction

Within the hippocampus (dentate gyrus, CA fields, and subiculum), the subiculum has a unique status. Unlike any other subfield, the subiculum projects to almost all external sites innervated by the hippocampus (O’Mara, 2005). In addition, some key hippocampal projections arise almost exclusively from the subiculum. Examples include the dense hippocampal efferents to the mammillary bodies (MBs), anterior thalamic nuclei, and retrosplenial cortex (RSP; areas 29, 30), which together form an extended limbic network (Rolls, 2015; Bubb et al., 2017). These limbic interconnections have been regarded as vital for emotion (Papez, 1937; MacLean, 1949; Dalgleish, 2004) and, more recently, for spatial memory in rodents and episodic memory in humans (Aggleton et al., 2010; Carlesimo et al., 2011; Ritchey et al., 2015). These same hippocampal connections are also directly implicated in the memory loss that characterizes the earliest stages of Alzheimer’s disease (Tan et al., 2013; Aggleton et al., 2016). Consequently, understanding the nature of these hippocampal connections remains a priority.

A feature of the projections from the various hippocampal CA fields is that they standardly collaterize to innervate multiple sites (Swanson et al., 1981; Donovan and Wyss, 1983). In contrast, projections from the subiculum are typically segregated by their columnar and laminar site of origin (Witter et al., 1990; Ishizuka, 2001; Witter, 2006; Christiansen et al., 2016). A consequence is that many subiculum neurons only innervate one target site (Swanson et al., 1981; Donovan and Wyss, 1983; Namura et al., 1994; Naber and Witter, 1998; Wright et al., 2010, 2013). There are, however, reasons to suppose that the hippocampal projections to the RSP and MBs might prove different, as populations of subiculum neurons that project to these two sites seem to be present in overlapping regions of the subiculum in both rats and monkeys (Van Groen and Wyss, 2003; Kobayashi and Amaral, 2007; Christiansen et al., 2016). For these reasons, the present study began by determining whether the source of these hippocampal projections was indeed from the same region of subiculum, before testing if these two sets of hippocampal efferents remain segregated or whether they provide collateral outputs to both targets. Resolving these issues is valuable as it has been presumed that the RSP and MBs are concerned with different aspects of hippocampal information processing (Byrne et al., 2007; Dillingham et al., 2015a). One potential basis for this difference would be if they derive information from separate hippocampal outputs.

The initial experiments, therefore, used multiple fluorescent tracers to determine whether the subiculum projections to the MBs and RSP arise from the same or different cell populations. One of the axonal tracers used in the present study, unconjugated cholera toxin B subunit (CTB), is transported in both anterograde and retrograde directions. A consequence is that “collateral-collateral” transport can occur (Chen and Aston-Jones, 1998). This form of transport occurs when a tracer is conveyed retrogradely in one collateral to reach the cell soma, where it is then conveyed anterogradely along other collaterals. This property not only makes it possible to specify the location of the particular collateral terminals under investigation, i.e., in either the MBs or RSP, but it also becomes possible to look for other collateral projections involving these same terminal sites. In follow-up experiments, surgical disconnections helped to test for whether collateral-collateral tracer transport from the hippocampus had, indeed, occurred. Those findings then led to more precise neurochemical characterizations of these shared limbic pathways.

## Materials and Methods

The principal experiments were performed on 34 adult, male Lister Hooded rats weighing 270–320 g (Envigo). Additional experiments involved two adult, male C57BL/6 mice weighing 32 and 35 g (bred at Cardiff University). Pairs of anatomical tracers were used in combination to allow double fluorescent labeling in the same animal. The fluorescent retrograde tracers fast blue (FB; Polysciences Inc), FluoroGold (Santa Cruz Biotechnology, Inc.), CTB-Alexa Fluor 488 (CTB_488_) and CTB-Alexa Fluor 594 (CTB_594_; Invitrogen). Additionally, unconjugated CTB (List Biological Laboratories Inc., product #103B) was used as it is transported along axons in both anterograde and retrograde directions. This tracer was visualized by immunofluorescence. The tracer pairings were as follows: FB + FG, *n* = 6; CTB_488_/CTB_594_ + FB, *n* = 4; CTB in MBs + FB in RSP, *n* = 5; FB in MB + CTB in RSP, *n* = 2. Single tracer studies using only CTB were also conducted: CTB in RSP, *n* = 3; CTB in MB only, *n* = 4. A final, additional set of two adult male Lister Hooded rats received injections of the anterograde tracer, 3-kDa biotinylated dextran amine (BDA; Life Technologies Ltd) in the dorsal hippocampus to provide additional information about the termination sites of possible collateral connections. All experiments were in accordance with United Kingdom Animals (Scientific Procedures) Act, 1986 and associated guidelines, and approved by local ethical committees at Cardiff University.

### Surgical methods, rats

All rats were anesthetized throughout surgery with isofluorane (5% for induction, 2% thereafter). Rats were placed in a stereotaxic frame (Kopf), with the mouth-bar set at +5.0mm. For analgesic purposes, Lidocaine was administered topically (0.1 ml of 20-mg/ml solution; B. Braun) and meloxicam was given subcutaneously (0.06 ml of 5-mg/ml solution, Boehringer Ingelheim Ltd). Under aseptic conditions, small openings were made in the skull and dura to allow access for a 0.5-µl Hamilton syringe for pressure injections (25 ga, Hamilton).

Single tracer injections (per hemisphere) were made in the MBs. The coordinates centered on anterior-posterior (AP) -1.9, medial-lateral (ML) ±0.5, and dorsal-ventral (DV) -10.4 from bregma, but varied slightly to encompass different subregions. For the RSP, six injections ensured coverage along the full AP plane of this large cortical area. The six coordinates, relative to bregma, with depth relative to top of cortex, were: AP −1.8, ML ±0.5, DV −1.0; AP −2.8, ML ±0.5, DV −1.0; AP −4.0, ML ±0.5, DV −1.0; AP −5.8, ML ±0.5, DV −2.5; AP −5.8, ML ±0.9, DV −1.4; AP-6.6, ML ±0.9, DV −1.8. Animals received either bilateral or unilateral injections in the same structure.

Unconjugated-CTB, CTB_488_, and CTB_594_ were made up as a 1% solution in sterile 0.1 M PBS (pH 7.4), FB was made up as a 3% solution in sterile PBS (pH 7.4), while FluoroGold was made up as a 4% solution in sterile, distilled water. Following pressure injections of 0.06–0.1 µl into each site, the syringe was left in place for at least 5 min to help reduce any back flow of the tracer. For the RSP, there was no concern about tracers traveling back up the syringe tract, however, some evidence of the tracers could be detected from the syringe tracks immediately above the MB injections.

For the anterograde tracer studies, BDA was made up as a 10% solution in sterile, distilled water (pH 7.4) and injections were made at three sites along the AP axis of the dorsal subiculum. The injection coordinates relative to bregma were: AP −4.4, ML ± 2.9, DV −5.8; AP −5.0, ML ± 3.8, DV −6.7; AP −5.3, ML ± 4.9, DV −8.3. Injection volumes were 0.06–0.08 µl. The pressure injections were made over 10 min with the syringe left in place for at least 5 min to help reduce back flow of the tracer.

After completion of the tracer injections, the scalp was sutured and animals received a 5-ml subcutaneous injection of 5% glucose in 0.9% saline (Baxter Healthcare Ltd). Clindamycin hydrochloride antibiotic powder (Fort Dodge Animal Health Ltd) was applied over the closed, sutured scalp. Animals recovered in a thermostatically controlled container before returning to individual housing with *ad libitum* food and water.

### Surgical methods, mice

The mice were anesthetized throughout surgery with isofluorane (5% for induction, 2% thereafter). Mice were placed in a stereotaxic frame using a flat skull orientation. Lidocaine was administered topically (0.1 ml of 2-mg/ml solution) and meloxicam was given subcutaneously (0.06 ml of 0.5-mg/ml solution). Under aseptic conditions, small openings were made in the skull and dura to allow access for a 5µl Hamilton syringe (33 ga) connected to a UMP3 microsyringe pump injector (World Precision Instruments) with a flow rate of 0.02 µl/min.

A single tracer injection (CTB, 0.05 µl) was made in the MBs with coordinates AP −2.1, ML +0.2, DV −5.5 from bregma. For the RSP, two ipsilateral FB injections (both 0.1 µl) ensured spread along the cortex. The coordinates, relative to bregma were: AP −1.5, ML ±0.2, DV −0.8; AP −2.4, ML ±0.2, DV −1.0. Postsurgical care was the same as for rats, except that the mice received a 0.5-ml subcutaneous injection of 5% glucose in 0.9% saline.

### Testing the collateral-collateral transport of CTB: fornix lesions

Surgical disconnections were used to test whether CTB injected into the MBs could first be transported retrogradely in the fornix to the hippocampus (subiculum), but then be transported anterogradely in the same subiculum neuron to the RSP (collateral-collateral transport). For this reason, in some rats, lesions were made in the fornix, followed by CTB tracer injection into the MBs. Although it was possible to conduct the complementary experiment, i.e., injecting CTB into RSP after fornix lesions, this procedure was not conducted as there are light, direct projections from RSP to the MBs (Van Groen and Wyss, 2003).

Bilateral radio frequency lesions were targeted at the postcommissural descending fornix (*n* = 4). This region of the fornix was the preferred target as it is the subdivision of the fornix taken by neurons projecting from the subiculum to the MBs (Swanson and Cowan, 1977). The lesions were made using a thermocouple radio frequency electrode (0.3-mm active tip length, 0.25 mm in diameter; Diros Technology Inc.). The electrode was lowered vertically and the tip temperature was then raised to 70–74°C for 45 s using an OWL Universal RF System URF-3AP lesion maker (Diros Technology Inc.). The stereotaxic coordinates from bregma were: AP −0.2, LM ±1.2, DV −8.4, with the mouth-bar set at + 5.0 mm.

### Postoperative processing

Following a postoperative period of seven days, the rats were deeply anesthetized with sodium pentobarbital (Euthatal, Merial). They were then perfused intracardially with 0.1 M PBS at room temperature followed by 4% paraformaldehyde in 0.1 M PBS. Brains were removed and postfixed in the dark for 4 h in paraformaldehyde and then transferred to 25% sucrose solution in 0.1 M PBS for 24 h in the dark before sectioning into 40-µm coronal sections with a freezing microtome (Leica 1400). A 1-in-4 series of sections was mounted directly onto gelatin-subbed slides and then allowed to dry at room temperature. This series was stained with cresyl violet to help localize the injection sites. For the surgical cases involving FB, FluoroGold, CTB_488_, or CTB_594_, a second 1-in-4 series was mounted directly onto gelatin-subbed slides, allowed to dry in the dark, dehydrated in increasing concentrations of alcohol, then coverslipped using DPX (Sigma-Aldrich).

For the cases involving CTB, the second tissue series was immunohistochemically stained for that tracer. The sections were incubated in a solution of rabbit-anti-cholera toxin primary antibody (1:10,000; Sigma-Aldrich, product #C3062, batch 104M4768V; RRID: AB_258833) and 1% normal goat serum in 0.1 M PBS for 24 h at room temperature. Following washing, the sections were incubated with DyLight 594, goat-anti-rabbit (1:200; Vector Laboratories, product #DI-1594; RRID: AB_2336413) for 24 h at 4°C. Sections were then mounted onto gelatin-subbed slides, allowed to dry, dehydrated in increasing concentrations of alcohol, and coverslipped with DPX.

For the cases involving BDA, the second tissue series was incubated in the Vectastain ABC solution (Vector Labs) for 2 h, then washed in PBST twice for 10 min each, followed by a further three washes in 0.1 M PBS. Sections were then reacted with diaminobenzidine (DAB; Vector Labs) and intensiﬁed with nickel, after which they were mounted, dried, and coverslipped, as described above.

Sections were viewed using a Leica DM5000B microscope for both transmitted white light (for sections stained with cresyl violet) and fluorescence microscopy (for sections with a fluorophore). An attached Leica DFC350FX digital camera and LAS AF image acquisition software (Leica) were used to capture high resolution images.

### Experimental design and statistical analysis

FB in conjunction with FluoroGold was used for initial qualitative analyses of the two pathways. For quantitative analyses, FB injections were paired with CTB injections into the MBs or RSP. The combination of FB and CTB was chosen for quantification as these tracers have distinctive emission wavelengths (420 and 618 nm, respectively) and fill neuronal cell bodies in different ways (Köbbert et al., 2000). Cell counts were only taken from those animals in which the respective injections were correctly located.

Double-labeled subicular neurons were counted using the object-based colocalization methods of Just Another Colocalization Plugin, a plugin to the public domain, ImageJ software (Bolte and Cordelières, 2006). This software allowed for the initial identification of subicular neurons that project to each region separately. The plugin then determined the fluorescence intensity centers of the CTB-positive subcellular structures and identified the locations at which they coincide with FB. The system was tested using images that were taken on the same microscope, under the same conditions as the images to be analyzed. These test images had either two overlapping (different fluorophores targeting the same protein) or nonoverlapping distributions of fluorescent staining. The colocalization analysis was conducted in four regions of interest across the proximal-distal axis of the dorsal subiculum (Christiansen et al., 2016). An average of ten dorsal subiculum sections from −5.16 to −6.60 mm posterior to bregma (Paxinos and Watson, 2005) were analyzed for each case. Cell counts were taken from the dorsal subiculum as this is the source of the hippocampal projections to RSP (Van Groen and Wyss, 2003).

### Postoperative processing: additional immunofluorescent targets

These analyses examined the sites of collateral-collateral transport termination. Selected targets followed inspection of the Allen Brain Atlas (http://www.brain-map.org). Accordingly, antibodies raised in mouse for calbindin D28k (1:10,000; Swant, product #300; RRID: AB_10000347), calretinin (1:5000; Swant, product #6B3; RRID: AB_10000320), cholecystokinin 8 (1 in 500; Abcam, product #ab37274; RRID: AB_726010), GAD67 (1:1000; Merck Millipore, product #MAB5406; RRID: AB_2278725), parvalbumin (PV; 1:15,000; Sigma-Aldrich, product #P3088; RRID: AB_477329), neurotensin (1:100; product #SAB4200703, Sigma-Aldrich), VGluT1 (1:300; product #ab193595, Abcam), and VGluT2 (1:300; product #ab7915, Abcam) were included. The secondary antibody, DyLight 488, horse-anti-mouse (1:200; Vector Laboratories, product #DI-2488; RRID: AB_2307439) was used for visualization. Processing followed standard protocols (Dillingham et al., 2015b). All antibodies were tested before use to help confirm regional specificity by reference back to the Allen Brain Atlas. Immunohistochemical analyses were conducted on series of tissue from a subset of the surgical cases described above; CTB in MB + FB in RSP, *n* = 4; FB in MB + CTB in RSP, *n* = 1; CTB in MB only, *n* = 4.

For the examples of the higher magnification (40×) images of VGluT2 and NT, Manders’ coefficient of colocalization was estimated, again using Just Another Colocalization Plugin (Bolte and Cordelières, 2006). The M_1_ coefficient quantifies the proportion of the green signal coincident with a signal in the red channel over its total intensity. This measure can fall between zero (no overlap) and one (complete colocalization).

### Anatomic nomenclature

Anatomic names and borders follow Swanson (1992), except for the divisions within the RSP and postsubiculum, which use the terminology of Van Groen and Wyss (2003). The latter authors divide RSP into a dorsal, dysgranular subregion (Rdg, area 30) and two ventral, granular subregions (Rga, Rgb, area 29). (Note, other authors further subdivide area 29, e.g., Jones and Witter, 2007.) Here, the rat subiculum is divided into two layers, i.e., a superficial molecular layer and a deeper, thick layer of pyramidal cells (Kloosterman et al., 2003). The term “intermediate subiculum” refers to that subiculum region at the caudal extent of the hippocampal flexure where the dorsal subiculum and ventral subiculum converge (Bast et al., 2009). In accordance with Witter and Wouterlood (2002), the subiculum is included within the hippocampus, while the presubiculum and parasubiculum (and postsubiculum) form parts of the parahippocampal region.

## Results

**Figure 1.**
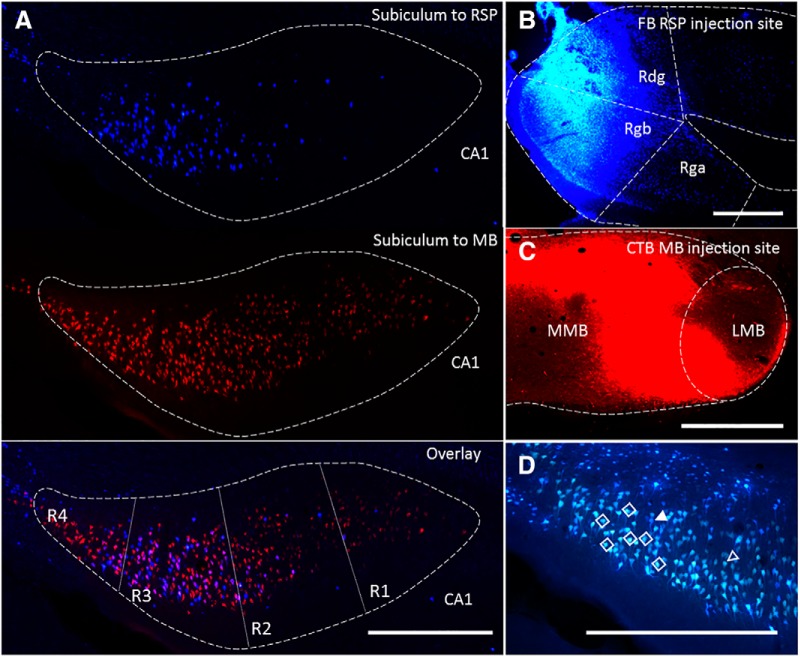
Subicular neurons collateralize to innervate the RSP and MBs. ***A***, Coronal photomicrographs of dorsal subiculum in a rat following FB injections in RSP and CTB in the MBs with pink double-labeled cells in the overlay panel indicating neurons that collateralize to both regions. Proximal-distal regions (R1–R4) were divisions used for subsequent quantification. ***B***, Coronal section showing FB injection into RSP. ***C***, Coronal section showing CTB injection into MBs. ***D***, Coronal dorsal subiculum section after injections of FB into the RSP and FluoroGold into the MBs. The open arrowhead points to a single-labeled neuron projecting to MB, the closed arrowhead to single-labeled neuron projecting to RSP, the open diamonds indicate double-labeled neurons. CA1, hippocampal field CA1; LMB, lateral mammillary nucleus; MMB, medial mammillary nucleus; Rga and Rgb, granular RSP, subdivisions a and b, respectively (collectively, area 29); Rdg, dysgranular RSP (area 30). Scale bars: 500 µm.

In an initial series (*n* = 3), injections of FB and FluoroGold helped to confirm the presence of overlapping populations of dorsal subiculum neurons that project to the two target regions (Fig. 1*D*). Within these overlapping populations of pyramidal cells (blue to RSP, yellow to MBs), some cream-colored cells were observed (Fig. 1*D*). These additional neurons are presumed to send axons to both the MBs and RSP. A similar pattern of results was obtained with the reverse tracer-target configuration (*n* = 3). This pattern was further corroborated using CTB conjugated to Alexa Fluors (CTB_488_ and CTB_594_), in combination with either FB or FluoroGold (*n* = 4).

**Figure 2.**
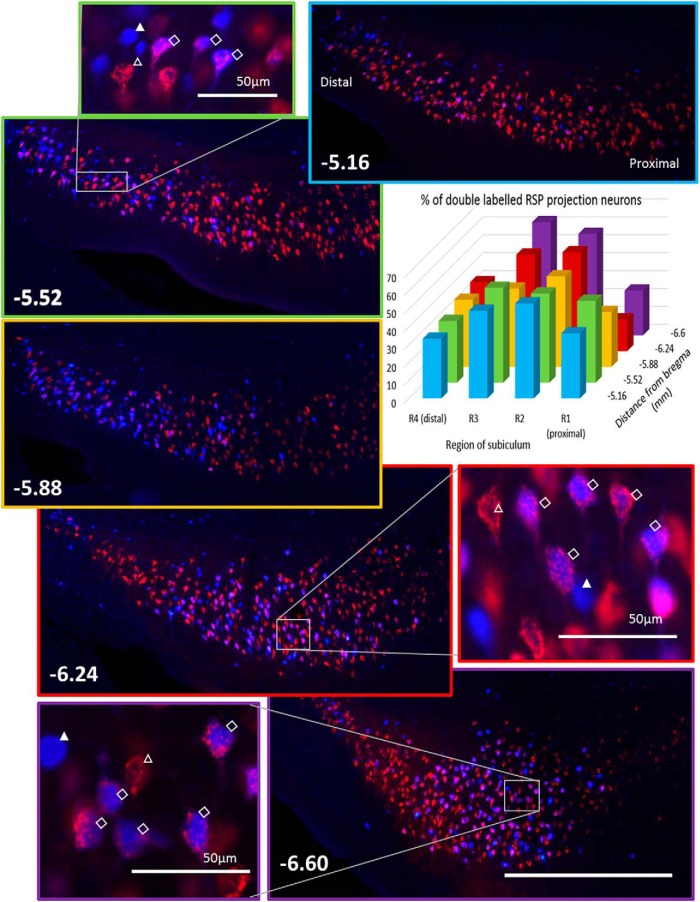
Quantification of extent and location of collateralizing neurons in dorsal subiculum. Histogram illustrates the percentage of subiculum neurons projecting to RSP that colabel with MB tracer. For this analysis, dorsal subiculum was divided by proximal-distal (R1–R4) and AP locations (cell counts are presented in Extended Data Fig. 2-1). Photomicrographs depict dorsal subiculum (right hemisphere) at five AP levels (numbers indicate distance from bregma in millimeters), the borders are color coded to match the corresponding bars in the histogram. The photomicrographs show pink double-labeled cells that innervate both sites, red neurons projecting to MB, and blue neurons projecting to RSP. Additional, higher magnification panels show labeling in more detail; FB (blue) fills the cytoplasm while retrogradely transported CTB (red) remains in vesicles and so appears granular. The open arrowhead marks a single-labeled neuron projecting to MB, the closed arrowhead marks a single-labeled neuron projecting to RSP, the open diamonds indicate double-labeled neurons. Scale bar: 500 µm unless otherwise specified.

To quantify this population of collateralizing projections more precisely, FB and CTB were separately injected into the two target sites (Fig. 1*B*,*C*). Of the acceptable injections, five cases involved CTB in the MBs and FB in RSP, while two rats received the reverse placement of tracers. Double-labeling was observed in pyramidal cells in the middle of Layer II of the septal and intermediate (dorsal) subiculum (Fig. 1*A*). The number of labeled neurons was estimated in four regions of interest along the proximal-distal axis of the subiculum (R1–R4; Fig. 2). Double-labeled neurons were most prevalent in the mid proximal-distal plane (R2 and R3) of the dorsal hippocampus (Figs. 1*A*, 2). The cell counts from these seven cases indicated that an overall mean of 46% (range, 41.8% to 64.3%) of the subiculum pyramidal neurons that project to the RSP also collateralize to innervate the MBs (Fig. 2; Extended Data Fig. 2-1). (This percentage is an underestimate as complete MB tracer uptake would be needed for a full count.) No apparent morphologic characteristics could be discerned to distinguish single from double-labeled cells.

10.1523/ENEURO.0383-17.2018.f2-1 Extended Data Figure 2-1 Numbers of CTB- and FB-positive cells within different proximal-distal positions (R1–R4) of the dorsal and intermediate subiculum of the rat, including the number of double-labelled cells. The case numbers and hemisphere of cell counts (R or L) are shown, along with the percentage of subicular cells projecting to the RSP that are double labelled. Download Figure 2-1, DOCX file.

**Figure 3.**
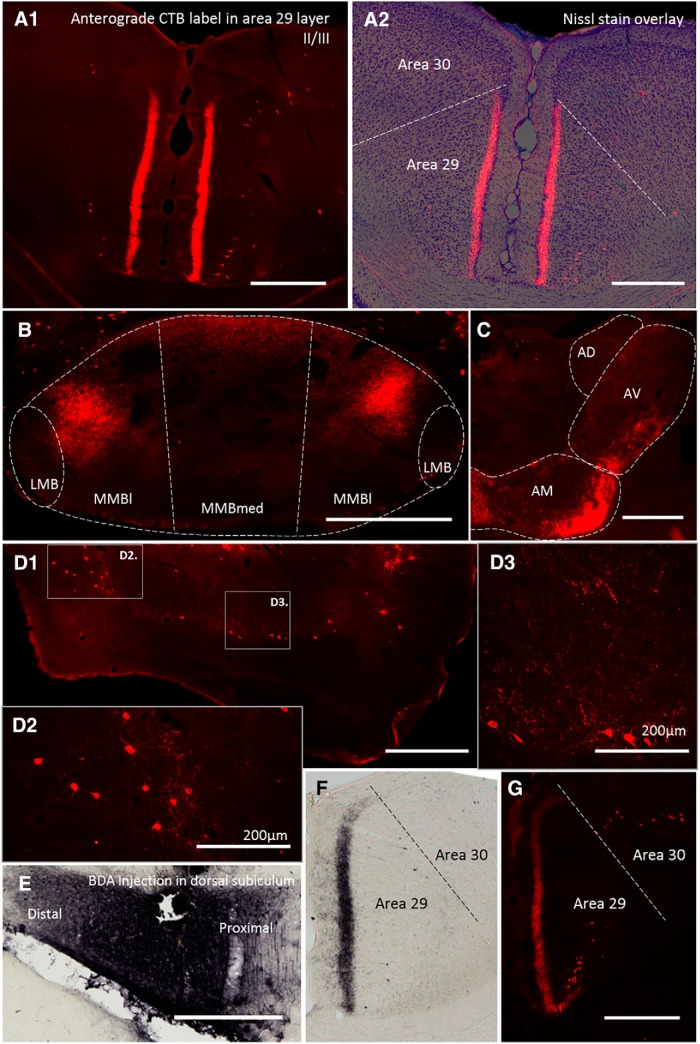
Characterization of collateral-collateral transport. ***A1***, Photomicrograph of collateral-collateral transport following a CTB injection into the MBs. The section shows CTB terminal label in Layers II and III of granular RSP (area 29). The Nissl-stained overlay (***A2***) confirms the abrupt border with dysgranular cortex (area 30). ***B***, Coronal section showing terminal label in dorsal pars lateralis (MMBl) and pars medianus (MMBmed) of the medial mammillary nucleus following a retrosplenial CTB injection. Note, pia artifact has been removed. ***C***, Coronal section showing dense terminal label in the anterior thalamic nuclei. ***D***, Pattern of both retrograde and light terminal label in the entorhinal cortex after a CTB injection into the MBs. Boxes, ***D2***, and ***D3*** correspond to higher magnification images of medial and lateral entorhinal cortex, respectively. ***E***, Photomicrograph of dorsal subiculum following injection of an anterograde tracer (BDA). ***F***, Coronal section of RSP showing pattern of BDA anterograde transport from dorsal subiculum. ***G***, Coronal section from same level of RSP as depicted in ***F***, illustrating pattern of CTB terminal label following CTB injection in MBs. AD, anterodorsal thalamic nucleus; AM, anteromedial thalamic nucleus; AV, anteroventral thalamic nucleus; LMB, lateral mammillary nucleus; MMBl, medial MB, pars lateralis; MMBm, medial MB, pars medialis. Scale bars: 500 µm unless otherwise specified.

After being transported retrogradely to the subiculum, CTB can travel anterogradely in the same neuron (Chen and Aston-Jones, 1998), labeling its collateral terminal fields (Fig. 3*A*,*B*). Consequently, four more rats received a CTB injection in the MBs, while three received CTB in the RSP. The MB CTB injections not only retrogradely labeled numerous cells in the subiculum of both hemispheres, but also produced a dense band of bilateral terminal label throughout deep Layer II and Layer III of granular RSP (Fig. 3*A*). This terminal label in areas 29a and 29b stopped abruptly at the border with dysgranular RSP (area 30). This pattern of terminal labeling matches that produced when an anterograde tracer such as BDA is injected into the dorsal subiculum (Fig. 3*E–G*), thus, is consistent with the direct projections from subiculum to RSP. Meanwhile, CTB injections in RSP led to ipsilateral, dorsal subiculum label, accompanied by (bilateral) terminal label in the medial mammillary nucleus, most evident in dorsal pars lateralis (Fig. 3*B*).

In those cases with CTB injections in the MBs, it was possible to look for anterograde label in other sites that do not receive direct mammillary inputs, as such label might reflect additional collateral connections. (The same procedure was not applied to those cases with CTB injections in RSP as, unlike the MBs, this cortical region innervates many different sites, so making interpretation more difficult.) As expected, dense anterograde label was observed in the anterior thalamic nuclei due to the very large projection via the mammillothalamic tract (Fig. 3*C*). Other sites containing terminal label included the prelimbic cortex, infralimbic cortex, the septum (medial and lateral), and the medial and lateral regions of entorhinal cortex (Fig. 3*D*). This entorhinal label was concentrated in the deep layers, predominantly in Layer V.

### Testing the collateral-collateral transport of CTB: fornix lesions

**Figure 4.**
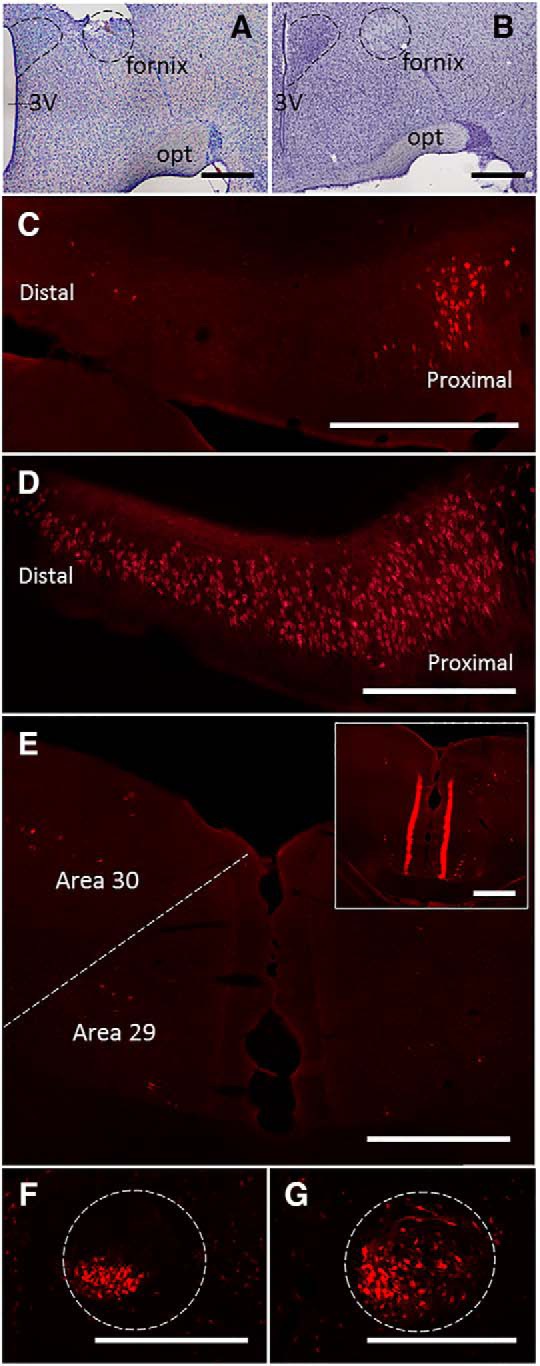
Absence of collateral-collateral transport to RSP following a CTB injection into the MBs combined with lesion involving the postcommissural descending fornix. ***A***, ***B***, Nissl-stained sections, 1.56 mm behind bregma (according to Paxinos and Watson, 2005), showing postcommissural fornix lesion (***A***) and intact case (***B***), respectively. ***C***, Coronal photomicrograph showing the very limited retrograde label in proximal dorsal subiculum after a postcommissural fornix lesion. ***D***, Typical appearance of retrograde label in the dorsal subiculum in an intact case (CTB in MBs). ***E***, Lack of terminal label in the RSP after postcommissural fornix lesion. The inset provides a comparison with an intact case. ***F***, ***G***, Retrogradely labeled neurons in Gudden’s ventral tegmental nucleus when the postcommissural descending fornix is lesioned (***F***) or intact (***G***) Note, while the label in ***F*** appears more restricted, it is denser. 3V, 3rd ventricle; opt, optic nerve. Scale bars: 500 µm.

In those cases with the most complete section of the postcommissural descending fornix (Fig. 4, compare *A*, *B*), the quantity of retrograde subiculum label was markedly attenuated after CTB injections in the MBs (Fig. 4*C*,*D*). In these cases (*n* = 2), the anterograde label in area 29 was no longer visible (Fig. 4*E*). This result, the elimination of terminal label in RSP, indicated that the anterograde label had originated via the subiculum inputs to the MBs. To confirm that this absence of tracer signal in the subiculum and RSP was not due to the tracer failing to be taken up by the MBs following fornix lesions, Gudden’s ventral tegmental nucleus was examined as this nucleus projects to the MBs, but not via the fornix (Allen and Hopkins, 1989). Comparable numbers of neurons labeled with CTB were observed in Gudden’s nucleus, whether the fornix had been cut or spared (Fig. 4*F*,*G*), confirming tracer uptake in both conditions.

### Cross-hemispheric collateral projections

The pattern of double and single labeling in the subiculum following tracer injections into one hemisphere indicated that the projections to the RSP remained ipsilateral to the subiculum while the collaterals to the MBs could arise from either the ipsilateral or contralateral subiculum.

### Cross-species comparisons

**Figure 5.**
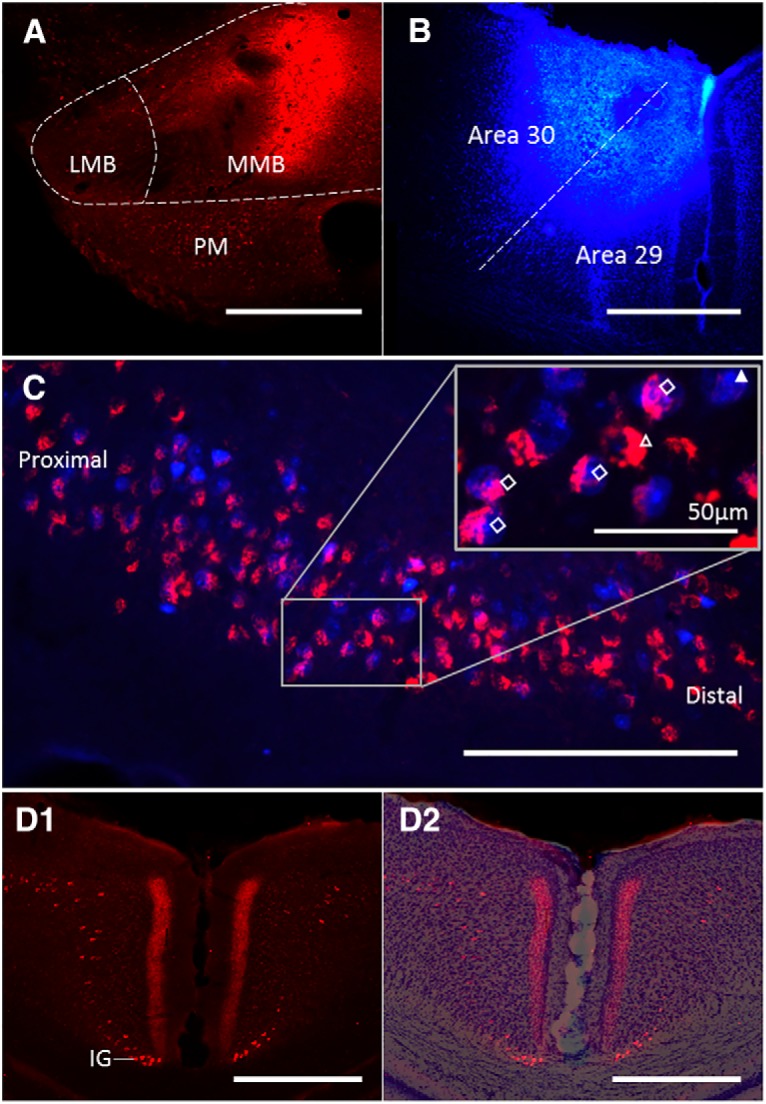
Cross-species comparisons. ***A***, Coronal section showing CTB injection into mouse MBs. ***B***, Coronal section showing FB injection into mouse RSP. ***C***, Coronal photomicrograph of dorsal subiculum. The numerous double-labeled (pink) cells innervate both sites. Inset depicts higher magnification of indicated region. The open arrowhead points to a single-labeled neuron projecting to MB, the closed arrowhead to a single-labeled neuron projecting to RSP, the open diamonds indicate double-labeled neurons. Associated cell counts are presented in Extended Data Figure 5-1. ***D1***, Red terminal label in the granular RSP (area 29) from collateral-collateral transport, alongside scattered retrogradely labeled cells in RSP and the indusium griseum (IG). ***D2***, A Nissl-stained overlay of section ***D1*** shows the border between area 29 and area 30. The label is concentrated in deep Layer II and Layer III of area 29. IG, indusium griseum; LMB, lateral MBs; MMB, medial MBs; PM, premammillary nucleus. Scale bar: 500 µm unless otherwise specified.

To determine whether these bifurcating subicular neurons are present in other rodents, the same anatomic methods were applied to adult mice (C57BL/6 strain). The tracer CTB was injected into the MBs (Fig. 5*A*) and FB injected into the RSP (Fig. 5*B*) generating a population of double-labeled neurons in the dorsal subiculum (Fig. 5*C*). Quantification of those subiculum neurons that project to RSP and also project to the MBs yielded remarkably similar results to those found in the rat (Extended Data Fig. 5-1). The colocalization analysis indicated that an overall mean of 41% of those subiculum neurons that project to RSP also collateralize to innervate the MBs (range across cases, 39.8–46.5%). Furthermore, CTB tracer injections in the MBs again resulted in dense terminal label, restricted to area 29 (Fig. 5*D*). This label was concentrated in deep Layer II and Layer III (Fig. 5*D*), consistent with collateral-collateral transport via the subiculum and the results seen in the rat.

10.1523/ENEURO.0383-17.2018.f5-1 Extended Data Figure 5-1 Numbers of CTB- and FB-positive cells within of the dorsal and intermediate subiculum of the mouse, including the number of double-labelled cells. The case numbers and hemisphere of cell counts (R or L) are shown, along with the percentage of subicular cells projecting to the RSP that are double labelled. Download Figure 5-1, DOCX file.

### Neurochemistry of subiculum efferents

**Figure 6.**
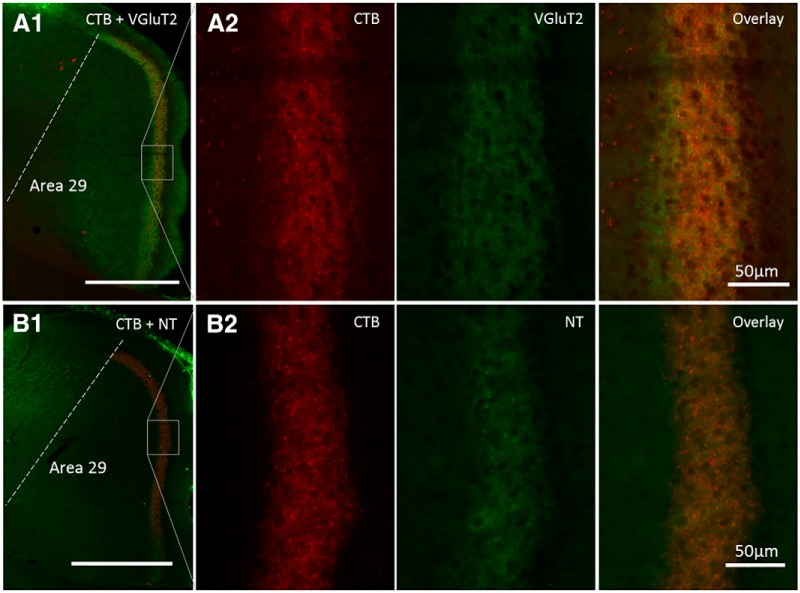
Neurochemical characterization of collateral-collateral terminals. ***A1***, Combined immunohistochemical signal for VGluT2 matching the distribution of CTB terminal label localized in superficial area 29. ***A2*** shows at greater magnification the separate CTB and VGluT2 label, with the overlay showing colocalization within Layers II and III of area 29. ***B1***, Combined immunohistochemical signal for neurotensin (NT) matching the distribution of CTB terminal label localized in superficial area 29. ***B2*** shows at greater magnification the separate CTB and NT label, with the overlay showing colocalization within Layers II and III of area 29. Scale bar: 500 µm unless otherwise specified. Note, pia artifact has been removed. Neurochemicals that did not colocalize with the CTB-positive terminals are shown in Extended Data Figure 6-1.

The ability to visualize the collateral projections within RSP made it possible to determine whether these subiculum efferents colocalize with specific neurochemicals. Using tissue from rats with CTB injections in the MBs, immunofluorescence revealed how the area 29 terminations specifically colocalized with signals for VGluT2 and neurotensin (Fig. 6*A*,*B*). This colocalization was very precise as both VGluT2 and neurotensin matched the CTB distribution in deep Layers II and III, but appeared absent from the rest of area 29. The colocalization in Figure 6 was estimated using Manders’ coefficient; for VGluT2 signal overlap with the CTB signal was M_1_ = 0.72, while for neurotensin the overlap with CTB was M_1_ = 0.96. Signals for neurotensin and VGluT2 were also present in dorsal pars lateralis of the medial MBs, i.e., those regions receiving collateral innervations. The CTB-positive area 29 terminations did not colocalize with VGluT1, GAD67, calretinin, PV, calbindin, or cholecystokinin (Extended Data Fig. 6-1).

10.1523/ENEURO.0383-17.2018.f6-1 Extended Data Figure 6-1 Series of coronal immunofluorescence images at the level of the RSP in an animal with a CTB injection in the MBs. Left column, Green immunofluorescent label associated with antibodies for VGluT1, GAD67, PV, calretinin (CR), calbindin (CB), and cholecystokinin (CCK). Middle column, CTB terminal label in the RSP (area 29, Layers II and III) highlighting the collateralizing subiculum projections that were present in the same section as depicted in the left column. Right column, The section overlay shows how the distribution of these neurochemicals do not match the termination sites of the collateral projections from the subiculum to area 29. Scale bar: 500 µm. Download Figure 6-1, TIF file.

As has been described previously (Varoqui et al., 2002), we found a paucity of VGluT1 label in deep Layer II and Layer III. GAD67 is a GABA-synthesizing enzyme and so was employed as a crude marker for GABAergic neurons to be followed up by other interneuron markers. GAD67 and CTB-positive terminals showed an almost complementary pattern of staining with GAD67 present in superficial Layer II and the deeper cortical layers but not deep Layers II and III (Extended Data Fig. 6-1). The pattern of PV labeling was, unsurprisingly, very similar to that of GAD67. Although nonoverlapping, there was a close association with CTB terminals in area 29 and PV-positive staining as PV cell bodies were found to sit among the CTB-positive terminals in deep Layer II and adjacent to PV-positive terminals in superficial Layer II (Extended Data Fig. 6-1); this pattern of PV staining matches previous descriptions (Salaj et al., 2015). Also consistent with previous reports (Salaj et al., 2015), calretinin had low but detectable levels of staining of both cells bodies and neuropil in RSP but there was a conspicuous absence of label in Layers II and III, and so no overlap with CTB. The final interneuron markers to be tested, calbindin and cholecystokinin, had very low levels of expression in RSP. Taken together, these results show that these CTB-labeled projections are excitatory rather than inhibitory.

## Discussion

**Figure 7.**
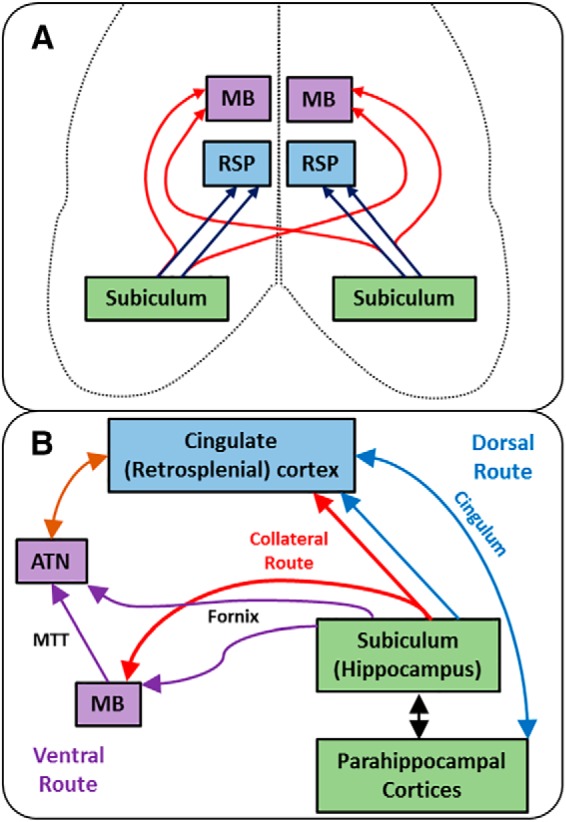
Schematic depictions of described hippocampal network connectivity. ***A***, Ipsilateral and crossed collaterals from the subiculum reach the MBs and RSP (area 29). Note, the subiculum projections to area 29 remain ipsilateral while collaterals to MB can remain ipsilateral or cross hemispheres. ***B***, Updated hippocampal-limbic network (Papez circuit) showing the ventral (subcortical), dorsal (cingulate), and new “collateral” routes. ATN, anterior thalamic nuclei; MTT, mammillothalamic tract.

The present study revealed collateral subiculum projections that simultaneously link the hippocampus with two sites, the MBs and the RSP (Figs. 1, 2). These shared projections arise from the dorsal subiculum, comprising almost half of the hippocampal projections to RSP in both rats and mice. For some of these collateral projections, the input from the subiculum to the MBs crosses to the opposite hemisphere (Fig. 7*A*). Meanwhile, the retrograde then anterograde movement of CTB, the latter via collateral-collateral transport, showed how the termination sites of these collateral projections are restricted to the medial mammillary nucleus and retrosplenial area 29 (Layers deep II and III; Fig. 3). Consequently, these two sites receive shared hippocampal information, despite the different contributions they make to learning and memory (Byrne et al., 2007; Vann et al., 2009; Dillingham et al., 2015a, Roy et al., 2017). This finding of a new category of subiculum neurons may relate to recent electrophysiological descriptions of multiple subpopulations of spatial cells within this same hippocampal region (Brotons-Mas, et al., 2017).

At the outset, it is important to confirm whether the CTB injections did, indeed, result in collateral-collateral transport, as such label best specifies the terminal sites of hippocampal collaterals within the RSP and MBs. The clearest evidence relates to the anterograde label observed in RSP following CTB injections into the MBs. First, there are no direct projections from the MBs to RSP (Van Groen and Wyss, 2003) and although transneuronal tracing has been observed using a biotin conjugate of CTB (Lai et al., 2015), unconjugated CTB is not thought to be trans-synaptically transported under the conditions used in the present study (Bilsland and Schiavo, 2009). While one potential trans-synaptic route would have been via the anterior thalamic nuclei, this would have principally produced anterograde label in Layers I and V of RSP (Shibata, 1993; Van Groen and Wyss, 2003). Instead, the observed label was restricted to Layers II and III. Second, the distribution of the retrosplenial terminal label precisely matched that of the direct projections from the subiculum to RSP (Fig. 3*F*; see also Van Groen and Wyss, 2003). Perhaps, most compelling, was the finding that surgical disconnection of the hippocampal projections to the MBs blocked the presence of this terminal label in RSP.

Evidence of transport of CTB from the RSP to the subiculum, and then to the medial MBs, was also observed, but this potential collateral-collateral label is more difficult to interpret. The difficulty arises because there is a very light, direct projection from granular RSP to the MBs (Van Groen and Wyss, 1990, 2003; see also retrograde labeled neurons in Fig. 3*A*). The apparent colocalization of the CTB label in the medial mammillary nucleus with neurotensin is consistent with this being collateral-collateral transport, but not proof. Likewise, the finding that the CTB label was concentrated in the dorsal medial mammillary nucleus is more consistent with a projection from the septal (dorsal) subiculum (Shibata, 1989; Kishi et al., 2000), especially as the sparse, direct retrosplenial inputs from Rga are scattered across the MBs (Van Groen and Wyss, 1990).

The collateral-collateral transport of CTB made it possible to look for other projections to the MBs that might collateralize, e.g., from the subiculum. The MBs lend themselves to this analysis as they only have a restricted set of efferent targets. Aside from the anterior thalamic nuclei, which receive especially dense, direct projections from the MBs, other sites containing terminal label included the medial and lateral regions of entorhinal cortex, as well as the infralimbic and prelimbic cortices. Of these sites, the entorhinal label is the most likely to reflect collateral-collateral connections via the subiculum as the other sites receive direct MB inputs (Hoover and Vertes, 2007). Furthermore, subiculum neurons that innervate both the MBs and entorhinal cortex have already been described (Donovan and Wyss, 1983; Roy et al., 2017). As the subiculum inputs to entorhinal cortex terminate in the deep layers (Sørensen and Shipley, 1979), this distribution is consistent with the present entorhinal terminal label reflecting collateral projections. It was, therefore, striking that the density of this terminal label in entorhinal cortex appeared far less than that seen in RSP (Fig. 3*A*,*D*), even when accounting for the more diffuse termination zone. Meanwhile, the value of appreciating hippocampal collateral projections has been highlighted by recent studies with mice. Roy et al. (2017) demonstrated the importance of subiculum neurons that collateralize to both the entorhinal cortex and MBs for fear memory retrieval (subiculum to entorhinal cortex) and for coincident fear states associated with fear memory retrieval (subiculum to MBs). They suggest that in their contextual fear conditioning paradigm the dorsal subiculum to MB projections regulate memory-retrieval-induced stress hormone responses, although it should be pointed out that the MBs have been implicated in many forms of spatial memory that do not involve an overtly stressful component (Vann and Aggleton, 2004; Vann and Nelson, 2015).

It should be added that the postsubiculum and regions of the medial prefrontal cortex also project to both MBs and RSP. Examination of these areas in our paired tracer studies revealed single labeled neurons but not double-labeled neurons. Thus, neurons in these regions are unlikely to contain neurons that collateralize to MBs and RSP.

The collateral-collateral transport of CTB also demonstrated the striking overlap between the collateral projections to area 29 and the presence of neurotensin and VGluT2, but not VGluT1. With known neurotensin projections from the subiculum to both the RSP and the MBs (Roberts et al., 1984; Kiyama et al., 1986), it now appears very likely that many of these same connections collaterize. Meanwhile, VGluT1 and VGluT2, which reflect different subclasses of glutamate terminal (Fremeau et al., 2004), occupy complementary areas within granular RSP (Varoqui et al., 2002). Their respective laminar locations within RSP are notable as they differ appreciably from that found across other cortical areas (Varoqui et al., 2002). Our tissue also indicates that the collateral subiculum projections to the MBs are again VGluT2 and neurotensin positive (Ziegler et al., 2002). Neurotensin can act as a neuromodulator to several neurotransmitter systems, including the glutamatergic system. A microdialysis study in freely moving rats demonstrated that neurotensin enhances cortical glutamate release, particularly by modulating the functional activity of cortical NMDA receptors (Ferraro et al., 2011). Thus, perhaps amplifying the excitatory signals from the hippocampus to these regions. While the analysis of these terminals permitted precise visualization of these subiculum-limbic efferents, it was not, however, possible to determine whether the collateral projections have properties that differ from those connections that only reach one target.

The present findings challenge notions about subiculum organization. Previous studies have shown that many subiculum connections are segregated by their columnar and laminar origin (Witter et al., 1990; Ishizuka, 2001; Witter, 2006; Wright et al., 2010, 2013; Christiansen et al., 2016), consequently subiculum neurons often innervate only one target. This property provides a marked contrast with the adjacent hippocampal CA fields (Swanson et al., 1981; Naber and Witter, 1998). The present findings now, however, show that the hippocampal (subiculum) inputs to the MBs may provide a special case as some of these inputs have collaterals to the retrosplenial cortices (present study) while, as others have already noted, there are also subiculum projections to the MBs with collaterals to the entorhinal cortex (Donovan and Wyss, 1983). In this way, subiculum neurons that collaterize link the hippocampus simultaneously with other sites that make different contributions to cognition (Vann et al., 2009; Todd and Bucci, 2015; Roy et al., 2017).

With respect to spatial processing, the MBs are closely linked with learning allocentric-based locations and providing head direction information, while the RSP is closely linked to landmark usage and changing reference frames (Vann and Aggleton, 2004; Byrne et al., 2007; Auger et al., 2012; Dillingham et al., 2015a; Vann and Nelson, 2015). RSP also contains cells coding for spatial context (Mao et al., 2017), as well as head direction cells linked to landmarks (Jacob et al., 2017). The mechanisms behind these complementary spatial functions become more tractable in light of the discovery of shared hippocampal projections to both sites. These same complementary features also highlight the key position of the anterior thalamic nuclei, which receive dense inputs from both the MBs and RSP, as well as the hippocampus. Consistent with this strategic location and the partial duplication of hippocampal inputs to the MBs and RSP, lesion studies in rats have shown that the anterior thalamic nuclei are more critical for hippocampal-sensitive spatial tasks than either the MBs or RSP (Aggleton et al., 1991, 1995; Neave et al., 1994). In addition, these thalamic nuclei show additional electrophysiological properties relating to spatial information (Tsanov et al., 2011; Jankowski et al., 2015) than either the MBs or RSP. These findings are consistent with the convergent involvement of the anterior thalamic nuclei in multiple aspects of spatial learning, which is partly fed by the collateral subiculum projections to the MBs and RSP.

The MBs, anterior thalamic nuclei, and RSP are key steps along a hippocampal return circuit (“Papez circuit”) historically presumed to be vital for emotion (Dalgleish, 2004; see Fig. 7*B*). These same sequential connections also provide the core of an extended hippocampal-limbic circuit, critical for episodic memory (Aggleton and Brown, 2006; Carlesimo et al., 2011; Rolls, 2015). The finding of a bifurcating pathway that allows the hippocampus to influence the diencephalon (MBs) and cingulate gyrus (RSP) either individually or in parallel (Fig. 7*B*), presents a different perspective. Indeed, in conjunction with other neuroanatomical studies (Jones and Witter, 2007; Kobayashi and Amaral, 2007), there is need to markedly revise this hippocampal-limbic circuit. Three parallel hippocampal-anterior thalamic routes emerge in this new account (Fig. 7*B*). First, a “ventral” subcortical route, via the fornix to the MBs and anterior thalamic nuclei, i.e., the original Papez circuit. Second, a “dorsal” cortical route, containing multiple two-way interconnections between the subiculum, RSP, and anterior thalamus (Bubb et al., 2017). Third, the new collateral pathway that unites both the ventral and dorsal routes. These findings create novel hippocampal networks for information processing in the thalamus, cingulate cortices, and beyond. These anatomic insights are timely as growing evidence links episodic memory loss in mild cognitive impairment and early Alzheimer’s disease with the breakdown of this same extended hippocampal network (Tan et al., 2013; Aggleton et al., 2016).

## Synthesis

Reviewing Editor: Menno Witter, Norwegian University of Science and Technology Decisions are customarily a result of the Reviewing Editor and the peer reviewers coming together and discussing their recommendations until a consensus is reached. When revisions are invited, a fact-based synthesis statement explaining their decision and outlining what is needed to prepare a revision will be listed below. The following reviewer(s) agreed to reveal their identity: Robert Vertes, Rebecca Burwell.

Advances the field

These findings are important in several ways. It's remarkable that such a large proportion of subicular cells target the medial mammillary nucleus and granular retrosplenial cortex. This in part runs counter to the prevailing view of the output of the subiculum, and the authors thus suggest this requires a revision of how we view an extended memory system, sometimes called the limbic memory system, that starts and ends with the hippocampal formation and includes anterior thalamus and cingulate cortex. We already knew that this network is more complex than the serial network originally described. However, I do agree that the identification of these bifurcating subicular neurons is an important finding that will help us understand how this circuitry supports spatial cognition. These findings will also help us understand the function of retrosplenial cortex about which there is much disagreement agreement. Finally, this study and this sort of work are also important because they contribute to the classification of pyramidal cells. Unlike interneurons, which can be classified on a number of dimensions, for example, morphology, axon lengths, physiological characteristics, and expression patterns, there are few dimensions on which pyramidal cells can be classified other than projection targets.

General

Unlike the rest of the hippocampal formation, the subiculum projects to the majority of cortical and subcortical hippocampal targets. Using various anatomical tracing techniques, the authors examined collateral projections from the dorsal subiculum to the retrosplenial cortex (RSP) and to the mammillary bodies (MB) in rats and mice. They demonstrated that a new class of dorsal subicular pyramidal neurons, forming approximately 50% of dorsal subicular cells, send bifurcating axons to the RSP and MB. This runs somewhat counter to the prevailing view that the subiculum generally distributes to single targets, which is in contrast to CA1 neurons that generally collateralize. The findings have direct implications for the role of this circuity in hippocampal-dependent memory functions. This circuitry is nicely depicted in Fig. 6.

The manuscript is clearly writing and the figures are good (but see comments below). The reviewers think the experiments are compelling and we can't think of another explanation for the results other than the authors' interpretations. The comparative work and the potential contribution to classification of principal neurons in subiculum are an added bonus. We however feel that the paper can be improved and require the authors to address the following points.

Comments and suggestions

1. In the Methods section it is important to indicate the number of rats (of a total of 32) that were used in each phase of the experiment. Although some of this information is contained in the Results section, it tends to get confusing and best to initially describe this in the Methods. For instance, it is unclear how many rats were used for post-processing for the identification of various neurochemical substances.

2. As mentioned, they reported that about 50% (or greater) of subicular cells retrogradely labeled with RSP injections were double labeled, indicating a very high percentage of collateral projections from the subiculum to the RSP and MB. While this has been carefully documented in Fig. 1D and Table 1, including percentages from the proximal to distal subiculum and at several A-P levels, this needs to be visually verified. Specifically, Fig 1-A2 or E show only isolated examples of double labeled cells; what is needed is a section(s) through the subiculum clearly showing substantial numbers of double labeled cells (> 50%) in a fairly large population of cells, preferably indicated by a distinct marker (arrow). In addition, the quality of the micrograph of Fig. 1-A2 showing double labeled cells could be improved or perhaps further magnified to clearly depict the two markers in the cells.

3. They used the retrograde and anterograde transport of CTB in a unique way to show that retrograde injections in MB gave rise to anterogradely labeled fibers in the RSP, termed collateral-collateral transport. While this is potentially a promising way to examine collateral projections from the subiculum to these sites, it is not without possible pitfalls. Specifically, whereas the subiculum is a major input to MB, the postsubiculum and regions of the medial prefrontal cortex also project to MB and could be the origin (or at least partially) of collateral projections to the RSP and MB - as examined with this method. The fornix lesions would seem to substantiate a role for the subiculum, but would they rule out the postsubiculum? This deserves comment. In addition, using this method the subicular projection to the RSP was very specific to layers II/III. It might be important to show this same pattern of labeling with anterograde injections (BDA or PHA-L) in the subiculum -- or their R2/R3 regions of the dorsal subiculum. Finally, they need to include micrographs showing the fornix lesions.

4. There is one paper suggesting CTB may be transneuronal (Lai et al, PLoS One. 2015). The authors should probably cite that paper and allay any concerns that these results might be confounded by transneuronal tracing.

5. To what extent did the anterograde labeling in MB with CTB injections in RSP result from anterograde projections from RSP to MB (which they acknowledge). Was there a way to determine this?

6. Apparently, the reason for using mice was to show the same pattern of projections in another rodent species. However, only two mice were used, and the same detailed analysis done for rats was not used for mice. We suggest adding some more information, including showing injection sites, and percentages of single/double labeled cells.

7.The section dealing with neurochemical markers would benefit from a slightly more in-depth description and illustration. Alternatively, you may consider to limit this to a description of VGluT2 and neurotensin terminals.

8. While the labeling in the entorhinal cortex (Fig. 2 D1 and D2) could have, as the authors indicated, resulted via a route through the subiculum, these micrographs need to be improved - at the present illumination little can be seen.

9. The extended data in Figure 5-1 and Table 1-1 do provide data that is integral to the paper. Figure 5-1 shows some of the negative results for the immunofluorescence, and Table 1-1 provides the cell counts for each animal. This is good information for a reader to have access to. However, the photomicrographs in Figure 5, panels A2 and B2 should be better. It's not possible to evaluate the colocalization very well. Perhaps higher magnification panels could be added.

10. The Roy et al. (2017) study in the Discussion is not well explained.

11. P4. Please provide the product number for unconjugated CTB

12. P6. The 33 gauge syringe used of injections seems large. Were there issues will tracer following up the syringe track? This should be discussed.

Statistics and Software

The authors used the ‘Just Another Co-localization Plugin’ to the public domain ImageJ. This software may provide a statistic for the extent of neurochemical colocalization. If so, they should probably report this. Likewise, the authors should report whether any testing of the software was performed.

## Author Response

Dear Prof. Witter

Thank you and the Referees for their very helpful comments. Below, we remind you of the revisions suggested (in italics), alongside an explanation of our revisions. The result has been an improvement in the clarity of the manuscript, while explaining better its rationale and findings. Changes to the manuscript have been highlighted within the document using red text.

1. In the Methods section it is important to indicate the number of rats (of a total of 32) that were used in each phase of the experiment. Although some of this information is contained in the Results section, it tends to get confusing and best to initially describe this in the Methods. For instance, it is unclear how many rats were used for post-processing for the identification of various neurochemical substances.

Additional information has been added to the first paragraph on page 5 that details the numbers of surgical cases of the various tracer locations and pairings. Also, further information has been included in the first paragraph of page 10 to give the numbers of rats, and their surgical history, with regard to the tissue used in post-processing neurochemical characterisation.

2. As mentioned, they reported that about 50% (or greater) of subicular cells retrogradely labeled with RSP injections were double labeled, indicating a very high percentage of collateral projections from the subiculum to the RSP and MB. While this has been carefully documented in Fig. 1D and Table 1, including percentages from the proximal to distal subiculum and at several A-P levels, this needs to be visually verified. Specifically, Fig 1-A2 or E show only isolated examples of double labeled cells; what is needed is a section(s) through the subiculum clearly showing substantial numbers of double labeled cells (< 50%) in a fairly large population of cells, preferably indicated by a distinct marker (arrow). In addition, the quality of the micrograph of Fig. 1-A2 showing double labeled cells could be improved or perhaps further magnified to clearly depict the two markers in the cells.

A new figure, Figure 2, has been added with micrographs of the dorsal subiculum at five A-P levels showing double labelled neurons. Additional higher magnification panels have also been included to illustrate single and double labelled neurons. These panels were taken at a higher magnification than the equivalent micrograph (Figure 1-A2) in the previous submission.

3. They used the retrograde and anterograde transport of CTB in a unique way to show that retrograde injections in MB gave rise to anterogradely labeled fibers in the RSP, termed collateral-collateral transport. While this is potentially a promising way to examine collateral projections from the subiculum to these sites, it is not without possible pitfalls. Specifically, whereas the subiculum is a major input to MB, the postsubiculum and regions of the medial prefrontal cortex also project to MB and could be the origin (or at least partially) of collateral projections to the RSP and MB - as examined with this method. The fornix lesions would seem to substantiate a role for the subiculum, but would they rule out the postsubiculum? This deserves comment. In addition, using this method the subicular projection to the RSP was very specific to layers II/III. It might be important to show this same pattern of labelling with anterograde injections (BDA or PHA-L) in the subiculum -- or their R2/R3 regions of the dorsal subiculum. Finally, they need to include micrographs showing the fornix lesions.

Examination of the postsubiculum and medial prefrontal cortex in our paired tracer studies revealed single labelled neurons but not double-labelled neurons. Thus, neurons in these regions are unlikely to contain neurons that collateralize to mammillary bodies and retrosplenial cortex. This point has been added to the Discussion on page 16.

An additional two surgical cases have been included in the paper. These rats received injections of the anterograde tracer, BDA, into the dorsal subiculum. The resultant anterograde label was specific to layers II/III of the retrosplenial cortex (RSP). Surgical details have been added to where appropriate in the Methods section and photomicrographs of an injection site and the anterograde label have been added to Figure 3 E, F.

A photomicrograph of the descending postcommissural fornix lesion, as well as an intact case for comparison, have been added to Figure 4 A, B.

4. There is one paper suggesting CTB may be transneuronal (Lai et al, PLoS One. 2015). The authors should probably cite that paper and allay any concerns that these results might be confounded by transneuronal tracing.

The Lai et al. (2015) paper has now been included in the Discussion on page 15 along with a description of the anterograde label that would be present, but was not observed, in RSP if the tracer had been transported trans-synaptically via the anterior thalamic nuclei, probably the main indirect route between MB and RSP.

5. To what extent did the anterograde labeling in MB with CTB injections in RSP result from anterograde projections from RSP to MB (which they acknowledge). Was there a way to determine this?

An additional paragraph to consider this point further has been added to the Discussion on page 15.

6. Apparently, the reason for using mice was to show the same pattern of projections in another rodent species. However, only two mice were used, and the same detailed analysis done for rats was not used for mice. We suggest adding some more information, including showing injection sites, and percentages of single/double labeled cells.

Micrographs of injection sites have been added to Figure 5. Additionally, a table detailing numbers of single and double-labelled neurons for both cases have been added in extended data Table 5-1.

7. The section dealing with neurochemical markers would benefit from a slightly more in-depth description and illustration. Alternatively, you may consider to limit this to a description of VGluT2 and neurotensin terminals.

A new paragraph has been added to the ‘Neurochemistry of subiculum efferents’ section (pages 13-14) that describes previously reported patterns of label along, with the patterns of labelling we observed for each of the neurochemical markers we visualised.

8. While the labeling in the entorhinal cortex (Fig. 2 D1 and D2) could have, as the authors indicated, resulted via a route through the subiculum, these micrographs need to be improved - at the present illumination little can be seen.

We apologise for the poor quality of the images in the previously submitted version of the manuscript, there seems to have been a problem when the figures were converted to pdf. The resolution of these images has been increased and an addition higher magnification panel of the entorhinal cortex has been included (Figure D2) to better illustrate this point.

9. The extended data in Figure 5-1 and Table 1-1 do provide data that is integral to the paper. Figure 5-1 shows some of the negative results for the immunofluorescence, and Table 1-1 provides the cell counts for each animal. This is good information for a reader to have access to. However, the photomicrographs in Figure 5, panels A2 and B2 should be better. It's not possible to evaluate the colocalization very well. Perhaps higher magnification panels could be added.

The structure of the neurochemical figure (now Figure 6) has been changed to include higher magnification panels of the red and green channels so that the individual patterns of cholera toxin B, neurotensin and VGluT2 positive staining can be evaluated and, thus, the colocalization can be better appreciated.

10. The Roy et al. (2017) study in the Discussion is not well explained.

We have expanded the description of the Roy et al. study and how it relates to the current work on page 16.

11. Please provide the product number for unconjugated CTB

Apologies for the omission, this has now been included in the first paragraph of page 5.

12. P6. The 33 gauge syringe used of injections seems large. Were there issues will tracer following up the syringe track? This should be discussed.

Due to the position of the retrosplenial cortex there was no issue with the tracers travelling back up the syringe tract, however, some evidence of the tracers could be detected along the syringe track following mammillary body injections. This has been added to the methods section on page 6.

The authors used the ‘Just Another Co-localization Plugin’ to the public domain ImageJ. This software may provide a statistic for the extent of neurochemical colocalization. If so, they should probably report this. Likewise, the authors should report whether any testing of the software was performed.

The co-localization software was tested using images that were taken on the same microscope, under the same conditions as the images to be analysed. These test images had either two overlapping (two different fluorophores targeting the same protein) or non-overlapping distributions of fluorescent staining. This point has been added to the methods section on page 9.

Co-localization between the neurochemical markers and cholera toxin B in Figure 6 has now been estimated by Manders' coefficient of colocalization, again using “Just Another Co-localization Plugin” (Bolte & Cordelières, 2006). The details have been added to Methods, page 6, and Results, page 14.
