# Supplementary material for: Collateral Projections Innervate the Mammillary Bodies and Retrosplenial Cortex: A New Category of Hippocampal Cells
Source: eNeuro. 2018 Mar 8;5(1):ENEURO.0383-17.2018. doi: 10.1523/ENEURO.0383-17.2018 (PMC5844061; doi:10.1523/ENEURO.0383-17.2018)
Supplement: Extended Data Figure 2-1 — Numbers of CTB- and FB-positive cells within different proximal-distal positions (R1–R4) of the dorsal and intermediate subiculum of the rat, including the number of double-labelled cells. The case numbers and hemisphere of cell counts (R or L) are shown, along with the percentage of subicular cells projecting to the RSP that are double labelled. Download Figure 2-1, DOCX file. [file sup_enu-eN-NWR-0383-17-s03.docx]

|  |  | ***Number of Subicular neurons*** | | | | |
| --- | --- | --- | --- | --- | --- | --- |
|  |  | **R1 (Proximal)** | **R2** | **R3** | **R4 (Distal)** | **Total** |
| ***#4R***  CTB in MB  FB in RSP | To MB | 423 | 657 | 1022 | 651 | 2753 |
|  | to RSP | 49 | 185 | 668 | 625 | 1527 |
|  | Double labelled | 11 | 82 | 372 | 224 | 689 |
|  | **% of double labelled RSP neurons** | **22.4** | **44.3** | **55.7** | **35.8** | **45.1** |
| ***#11L***  FB in MB  CTB in RSP | To MB | 747 | 1203 | 963 | 456 | 3369 |
|  | to RSP | 57 | 121 | 154 | 80 | 412 |
|  | Double labelled | 28 | 109 | 96 | 32 | 265 |
|  | **% of double labelled RSP neurons** | **49.1** | **90.1** | **62.3** | **40.0** | **64.3** |
| ***#11R***  FB in MB  CTB in RSP | To MB | 495 | 935 | 868 | 512 | 2810 |
|  | to RSP | 420 | 716 | 856 | 828 | 2820 |
|  | Double labelled | 148 | 383 | 425 | 274 | 1230 |
|  | **% of double labelled RSP neurons** | **35.2** | **53.5** | **49.6** | **33.1** | **43.6** |
| ***#12L***  CTB in MB  FB in RSP | To MB | 520 | 1327 | 973 | 596 | 3416 |
|  | to RSP | 21 | 165 | 290 | 206 | 682 |
|  | Double labelled | 2 | 97 | 186 | 54 | 339 |
|  | **% of double labelled RSP neurons** | **9.5** | **58.8** | **64.1** | **26.2** | **49.7** |
| ***#17L***  CTB in MB  FB in RSP | To MB | 1266 | 725 | 760 | 563 | 3314 |
|  | to RSP | 22 | 23 | 108 | 144 | 297 |
|  | Double labelled | 9 | 10 | 49 | 60 | 128 |
|  | **% of double labelled RSP neurons** | **40.9** | **43.5** | **45.4** | **41.7** | **43.1** |
| ***#17R***  CTB in MB  FB in RSP | To MB | 1193 | 1030 | 1068 | 530 | 3821 |
|  | to RSP | 25 | 320 | 710 | 445 | 1500 |
|  | Double labelled | 4 | 169 | 376 | 132 | 681 |
|  | **% of double labelled RSP neurons** | **16.0** | **52.8** | **53.0** | **29.7** | **45.4** |
| ***#20L***  CTB in MB  FB in RSP | To MB | 423 | 657 | 1022 | 651 | 2753 |
|  | to RSP | 49 | 185 | 668 | 625 | 1527 |
|  | Double labelled | 11 | 82 | 372 | 224 | 689 |
|  | **% of double labelled RSP neurons** | **8.9** | **39.4** | **53.0** | **37.2** | **41.8** |
