# Supplementary figures and images for: Collateral Projections Innervate the Mammillary Bodies and Retrosplenial Cortex: A New Category of Hippocampal Cells
Source: eNeuro. 2018 Mar 8;5(1):ENEURO.0383-17.2018. doi: 10.1523/ENEURO.0383-17.2018 (PMC5844061; doi:10.1523/ENEURO.0383-17.2018)

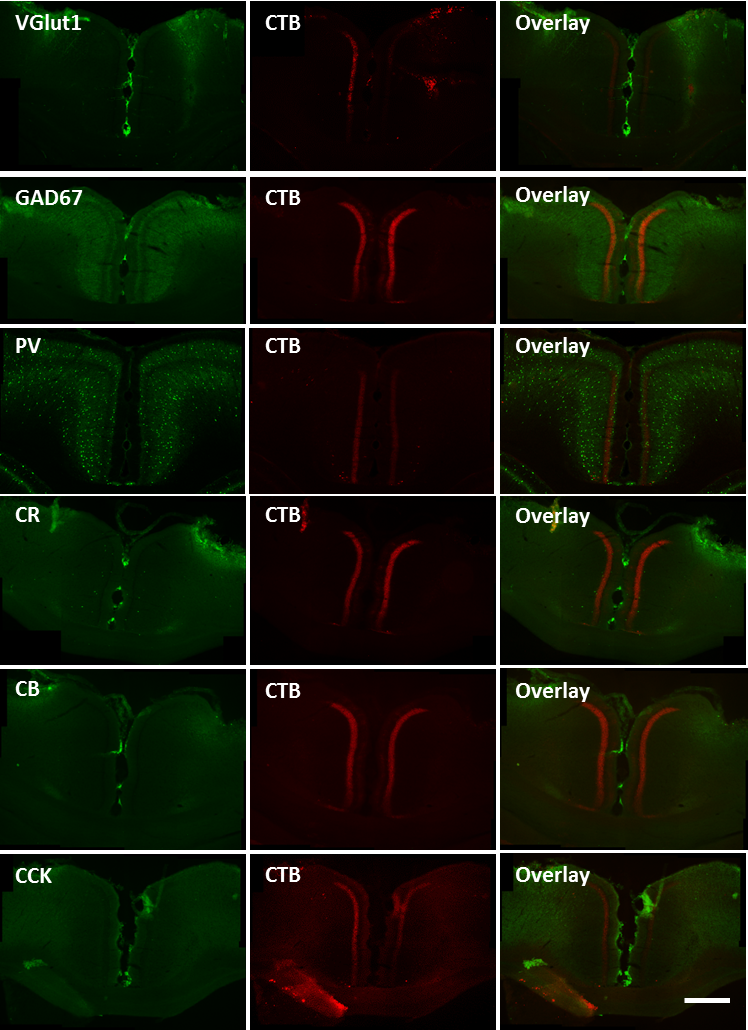

Supplement: Extended Data Figure 6-1 — Series of coronal immunofluorescence images at the level of the RSP in an animal with a CTB injection in the MBs. Left column, Green immunofluorescent label associated with antibodies for VGluT1, GAD67, PV, calretinin (CR), calbindin (CB), and cholecystokinin (CCK). Middle column, CTB terminal label in the RSP (area 29, Layers II and III) highlighting the collateralizing subiculum projections that were present in the same section as depicted in the left column. Right column, The section overlay shows how the distribution of these neurochemicals do not match the termination sites of the collateral projections from the subiculum to area 29. Scale bar: 500 µm. Download Figure 6-1, TIF file. [file sup_enu-eN-NWR-0383-17-s05.tif]
